# Promoting the adoption of local governmental policy on the reimbursement of chronic disease medicines (PAPMed): study protocol of a field-based cluster randomized trial in rural Nantong, China

**DOI:** 10.1186/s13063-022-06710-1

**Published:** 2022-09-15

**Authors:** Zhengting He, Xin Cao, Duan Zhao, Zemin Tang, Jiayu Zhao, Mariel Beasley, Angela Renne, Lei Liu, Shengjie Zhu, Yuexia Gao, Lijing L. Yan

**Affiliations:** 1 Global Heath Research Center, Duke Kunshan University, No.8 Duke Avenue, Kunshan, Jiangsu Province 215316 China; 2 Department of Epidemiology, Bloomberg School of Public Health, Johns Hopkins University, 615 North Wolfe Street, Baltimore, MD 21205 USA; 3 School of Public Health, Nantong University, No.9 Seyuan Road, Nantong, Jiangsu Province 226019 China; 4 School of Public Health, LKS Faculty of Medicine, The University of Hong Kong, No.7 Sassoon Road, Pokfulam, Hong Kong; 5 Center for Advanced Hindsight, Duke University, No.334 Blackwall Street, Durham, NC 27701 USA; 6 Duke University, Durham, NC 27708 USA; 7 Department of Gerontology, The First People’s Hospital of Kunshan, 91 Qianjin West Road, Kunshan, Jiangsu Province 215300 China; 8 School of Health Sciences, Wuhan University, No. 115 Donghu Road, Wuhan, Hubei Province 430072 China; 9 Duke Global Health Institute, Duke University, 310 Trent Drive, Durham, NC 27710 USA

**Keywords:** Non-communicable disease, Medication reimbursement policy, Rural health, China, Behavior science, Cluster randomized trial

## Abstract

**Background:**

Among rural Chinese patients with non-communicable diseases (NCDs), low socioeconomic status increases the risk of developing NCDs and associated financial burdens in paying for medicines and treatments. Despite the chronic disease medicine reimbursement policy of the local government in Nantong City, China, various barriers prevent patients from registering for and benefitting from the policy. This study aims to develop a behavior science-based intervention program for promoting the adoption of the policy and to evaluate the effectiveness of the program compared with usual practices.

**Methods:**

Barriers and opportunities affecting stakeholders in adopting the policy were identified through contextual research and summarized through behavior mapping. The intervention is designed to target these barriers and opportunities through behavior science theories and will be evaluated through a 6-month cluster randomized controlled trial in Tongzhou District, Nantong, China. A total of 30 villages from two townships are randomized in a 1:1 ratio to either the intervention or the control arm (usual practices). Village doctors in the intervention arm (1) receive systematic training on policy details, registration procedures, and intervention protocol, (2) promote the policy and encourage registration, (3) follow up with patients in the first, third, and sixth months after the intervention, and (4) receive financial incentives based on performance. The primary outcome is policy registration rate and the secondary outcomes include the number of patients registering for the policy, medical costs saved, frequency of village doctor visits, and health measures such as blood pressure and glucose levels.

**Discussion:**

This study is one of very few that aims to promote adoption of NCDs outpatient medication reimbursement policies, and the first study to evaluate the impact of these policies on patients’ financial and physical wellbeing in China. The simple, feasible, and scalable intervention is designed based on the theories of behavior science and is applicable to similar low-income regions nationwide where outpatient medical costs remain a financial burden for patients.

**Trial registration:**

ClinicalTrials.govNCT04731194, registered on 29 January 2021; Chinese Clinical Trial Registry ChiCTR2100042152, registered on 14 January 14 2021.

**Supplementary Information:**

The online version contains supplementary material available at 10.1186/s13063-022-06710-1.

## Background

In the past few decades, China has undergone tremendous socioeconomic development and demographic and epidemiologic transitions, characterized by changes in diet and lifestyles among an aging population. This consequently led to the shift from infectious diseases to chronic non-communicable diseases (NCDs) as the major causes of morbidity and mortality [1–3]. The number of deaths due to cardiovascular diseases, major complications of hypertension and diabetes, accounted for 46.75% of the total death of Chinese rural residents and 44.26% of the total death of Chinese urban residents in 2019, respectively [4]. To address the increasing NCDs burden, the central government issued a series of nationwide community-based strategies for NCDs prevention and control, including financial and administrative support, the use of community health centers in NCDs detection, diagnosis, treatment, and healthy lifestyle promotion [5, 6]. Most of these programs have been delivered to primary healthcare providers; however, there is no systematic strategy for monitoring practices for NCDs cases management at a grassroots level, currently [7, 8].

Rural residents constitute approximately 40% of the Chinese population [4] and have a greater hypertension and diabetes burden than in urban areas, due to increased prevalence but lower awareness, treatment, and control [9, 10]. The hypertension prevalence was estimated as 41.9% among 22,722 rural participants between 2005 and 2009 [11], and 46.2% in rural areas among 1.7 million adults screened from 2014 to 2017 in China [12]. The prevalence estimate of diabetes was 12.0% among rural residents in nationally representative cross-sectional survey from 2015 to 2017, according to diagnostic criteria from the American Diabetes Association [13]. Additionally, people with chronic diseases in rural areas were more likely to suffer from poverty than those in urban areas [14]. The economic burden caused by both hypertension and diabetes remains heavy, especially for rural residents.

The Residents-based Basic Health Insurance Schemes (RBHIS), established since 2009, were designed to prioritize inpatient services, leaving outpatient costs for hypertension and diabetes not covered or partially covered in most areas of China [15, 16]. Since 2019, the Chinese central government began to issue a series of RBHIS policy guidelines focusing on reducing outpatient costs for urban and rural patients with hypertension and diabetes [17]. Following the national guideline, several local chronic disease reimbursement schemes have been simultaneously established in many Chinese cities [18, 19]. By the end of 2019, Nantong City in Jiangsu province initiated an RBHIS reimbursement policy reform and improved hypertension and diabetes outpatient cost coverage. Before the RBHIS reform, outpatient costs for hypertension or diabetes were reimbursed only 400 Ren Min Bi (RMB) in a financial year. The new policy covers outpatient medication costs up to 50% or 1600 RMB per person for patients with hypertension or diabetes and 2000 RMB per person for patients with both diseases annually (essentials of the policy can be found in Additional file 1) [20, 21]. The policy applies to all registered hypertension and diabetes patients upon visiting designated public healthcare institutions. In order to qualify these outpatient benefit packages, hypertension or diabetes patients who are previously diagnosed, recorded in the chronic disease management system (CDMS), and continuously taking anti-hypertensive or anti-diabetic medications for treatment can register for the policy through the village clinics, township health centers (THCs), or secondary hospitals, while new patients have to be diagnosed with hypertension or diabetes and registered for the policy through a secondary hospital or above.

Unfortunately, most rural residents with NCDs were uninformed about this reimbursement scheme, which limited the effectiveness of this policy in reducing financial burdens of patients. Even patients who were aware of the policy may not be able to take advantage of the policy due to limited information on its registration process and details. It is well-known that engaging community health workers (CHWs) in primary healthcare is a cost-effective way to prevent and control NCDs, alleviate disease burden and mitigate resource shortage [6, 22–24]. Village doctors are main workforce of CHWs in rural areas of China, providing essential public health services within the National Essential Public Health System (NEPHS), including NCDs management for rural patients. Designing a theory-based intervention program to target the behavior of village doctors may provide opportunities to cost-effectively change the behavior of village doctors and rural patients in policy adoption and acquiring medication reimbursement, and thus alleviate financial burden and increase medication adherence of rural patients.

To effectively design theory-based behavior change interventions, it is important to identify the barriers and opportunities that influence the decisions of these stakeholders by two types: high-order cognitions (intrinsic personal values and norms) and low-order mental processes (external context and environment of the action) [25, 26], both of which are associated with CHWs engagement in NCDs prevention and control [6, 27, 28]. Theory-based intervention has proven to be effective in changing behavior in health intervention studies [29, 30], by targeting these barriers and opportunities with two approaches: by influencing high-order cognitions such as traditional health education [6, 28] and low-order mental processes such as financial rewards [26, 31, 32]. However, few intervention studies have been carried out in promoting health policy implementations in China to date.

To save medical costs for rural patients with hypertension and/or diabetes and to increase their medication adherence, we developed a simple, feasible, and scalable behavior science-based intervention program to promote the adoption of local governmental policy on the reimbursement of chronic disease medicines (PAPMed), currently being implemented and evaluated through a 6-month cluster randomized controlled trial. This paper describes the contextual research to inform the development of the intervention design and the research protocol of the study.

### Objectives

Our hypothesis is that the PAPMed intervention, compared to usual practices, can increase policy registration, save patient medication costs, and improve medication adherence and health outcomes. The primary objective of this study is to determine if the intervention program is superior to usual practices in promoting registration rate of rural patients in local governmental policy on outpatient reimbursement of chronic disease medicines. The secondary objectives are to determine if the intervention program is effective in reducing medication costs, increasing medication adherence, and improving health outcomes measured by blood pressure and blood glucose levels.

## Methods

The PAPMed trial is a cluster randomized controlled trial implementing in a rural area of Tongzhou District, in Nantong City, Jiangsu Province, China. The trial was registered in ClinicalTrials.gov (NCT04731194) as well as Chinese Clinical Trial Registry (ChiCTR2100042152) (details of the registration can be found in Additional file 2). The study protocol received ethics approval from the institutional review boards at Duke Kunshan University. Written informed consent (Additional file 3) will be collected from all participating intervention implementors prior to trial implementation. Reporting of the protocol adheres to the SPIRIT (Standard Protocol Items: Recommendations for Interventional Trials) guideline (Additional file 4) [33, 34]. Results of the trial will be published by principal researchers in the PAPMed trial in a peer-review journal. Any modifications to the protocol will be described in the final report of the trial results.

### Study site

This study is being conducted in Tongzhou District, which is a district with high NCDs burden. According to a health census report based on medical examination data of regional health records of 125,769 participants aged over 65, the overall prevalence of hypertension and type 2 diabetes were 53.8% and 12.1%, while the control rates were 41.0% and 47.0%, respectively in 2017 [35]. Despite rapid urbanization in the past decade, a large socioeconomic gap remains between rural and urban residents of the district. In 2019, the annual disposable income of rural residents was 25,846 RMB (about 4011 United States Dollars (USD)), less than half of that of urban residents in Tongzhou district [36].

Tongzhou transferred from a rural county to an urban district in 2009 with a large proportion of the residents remaining rural. Rural areas are consisted of 12 townships, with 205 villages under these townships [37]. Two townships were selected to take part in this study based on the selection criteria of having (i) a typical representation of the socioeconomic development of rural areas of Tongzhou District, (ii) with village clinics serving 400 NCDs patients or more per year; (iii) available village doctors who can be trained to implement the intervention, and (iv) support from local healthcare institutions and township leaders. Majority of the residents in these two townships are under rural *hukou* (Chinese household registration system).

The local healthcare infrastructure is consistent with the primary healthcare system of rural China, characterized by a hierarchical system of county hospitals, district health centers (DHCs), THCs, and village clinics [38]. DHCs are secondary-level hospitals responsible for diagnosing and treating NCDs patients. There is one village clinic in each village with 3 to 7 village doctors carrying out regular quarterly follow-up visits on NCDs patients under the NEPHS program, mainly including monitoring blood pressure and blood glucose, health education, and medication consultation.

### Contextual research to inform intervention design

#### Pre-intervention assessment

Before the main trial, a series of surveys and interviews were conducted to get insights into key challenges faced by stakeholders on policy implementation. The in-depth interviews were conducted with village doctors and NCDs patients in two randomly selected villages in each township. The interviews focused on the content and implementation of the policy, as well as the medication costs and compliance of patients. The research team further discussed opportunities and barriers of policy implementation and possible approaches to promote policy adoption with officials responsible for medical insurance in DHCs and the city medical insurance bureau. Furthermore, 153 village doctors in these two townships were invited to participate in a survey on their work of regular management of NCDs patients, awareness of and attitudes toward the policy, and willingness to participate in the policy promotion.

#### Current situations, identified opportunities and barriers

Table 1 shows a summary of findings from the in-depth interviews with patients, village doctors, and medical insurance officials, as well as a survey with village doctors. Most patients chose to purchase medications in public primary healthcare institutions, while pharmacies remained an option for a substantial group of patients, especially those near urban areas. Medication type, price, and convenience influenced the decision on medication purchasing locations. Financial burden was substantial especially among diabetic patients, mainly due to usage of insulin and comorbidity with hypertension. Medication non-adherence was prevalent, especially among young patients. Reasons for non-adherence were mainly attributed to forgetfulness and attitudes toward diseases and medications.

**Table 1:** Summary of findings from pre-intervention assessment with patients, village doctors, and medical insurance officials

Key themes	Interviewees	Main findings
**Medication costs and compliance**		
Medication purchase location	Patients, VDs	The most common place to buy medication is village clinics, followed by DHCs, pharmacies, secondary hospital or above.
		Most patients living near urban areas purchase medications in pharmacies due to lower price and more types of medications available.
		Most patients living near rural areas purchase medications in village clinics for convenience and lower price for some types of medications.
		Unavailability of certain medication types in village clinics. (This actually reflects the low awareness of policy details among patients, since policy applies to all public healthcare institutions, which have a great many type of medications available).
Medication costs	Patients	Hypertensive patients spend 30 to 50 RMB per month on self-medication, which is a low financial burden.
		Diabetic patients spend 200 to 300 RMB per month on self-medication, which is a heavy financial burden, and mainly due to the usage of insulin.
	Officials	Most patients with diabetes are also diagnosed with hypertension, adding up to the financial burden of self-medication.
Medication compliance	VDs	All interviewed VDs reported cases of medication self-discontinuation, especially among young patients, and attribute the situations to forgetfulness and insufficient awareness of comorbidity threats.
**Policy content and implementation**		
Policy awareness and implementation	Officials	Registration rates in both townships are low. About 12 and 2% of patients recorded in the hospital information system registered in the policy in the two townships, at the time of the interviews.
		Poor awareness on the policy details, both among officials of DHCs and village doctors.
		Low motivation to promote the policy among village doctors, at primary healthcare facilities.
	VDs	Most VDs have heard about the policy, but do not know details and do not know how to register.
		Among those VDs who know how to register, they only know patients can register in secondary hospitals and DHCs, but do not know they can help patients register just within village clinics.
		Many elderly VDs are not capable of operating the registration system.
		Operation systems on computers in some village clinics are outdated, and cannot install and run the registration system.
		Village doctors treat low awareness of the policy of the as the primary barrier for policy implementation, followed by complicated registration procedure, negative attitudes toward diseases and medications among some patients, inconvenient to buy medications in designated medical institutions, and low reimbursement rate.
	Patients	Majority of patients had never heard of the policy.
		Nearly all patients who have registered in the policy are those who visited the DHCs and registered by physicians in these DHCs. Even among these registered patients, some do not know they can enjoy the reimbursements provided by the policy.
Attitudes toward policy	Patients, VDs	Most patients and VDs think the policy can reduce financial burden and enhance medication adherence among patients.
		Several patients think the reimbursement rate provided is somewhat low.
	VDs	There are some patients heard about the policy from other registered patients and initiatively consult the policy with VDs.
Attitudes toward promoting the policy	VDs	All interviewed VDs would like to promote the policy to patients. The most common reasons for willing to promotion is to reduce financial burden for patients, followed by increasing patients follow-up rate, and requirements from supervisors.
		General self-efficacy on policy promotion is high.
		Some VDs are cautious toward large-scale promotion for increasing workload.
	Officials	Medical insurance staffs at DHCs are cautious toward large-scale policy promotion, since they may become overextended if large-scale registration takes place.

*Abbreviations*: *VDs* village doctors, *DHCs* district healthcare centers

Findings in the table are based on survey results from 153 village doctors; in-depth interviews with village doctors and patients in 4 villages, with 3–4 village doctors per village, as well as in-depth interviews with medical insurance officials during pre-intervention assessment

Our interview also found a low level of awareness of policy details among both patients and village doctors, in both townships, currently (Table 1). Only 12 and 2% of patients recorded in the CDMS were registered in the policy in the two participating townships, at the time of the interviews. Majority of interviewed patients had never heard of the policy. Most village doctors were unaware of details of the policy, as well as the registration process. Most patients and village doctors held positive attitudes toward the policy on reducing medical costs for patients, increasing follow-up and medication adherence rates. Barriers affecting the registration rate included low awareness, complicated procedures, and concerns about convenience of purchasing medications in designated medical institutions.

While most village doctors expressed willingness toward policy promotion and showed high self-efficacy in policy promotion (Table 1), they were cautious toward large-scale promotion. Most village doctors lacked the motivation to promote the policy since they regarded it as not within the scope of their responsibilities. Medical insurance staff at DHCs were also cautious toward large-scale promotion, since they may be overextended if large-scale registration takes place.

#### Behavior mapping

Evolved from the field of service design, the method of journey mapping provides pictorial illustrations of the experience in complex processes or interactions from an individual’s perspective regarding their relationship with surrounding organizations and services [39–41]. We modified journey mapping to develop the behavior mapping method to reflect the critical touchpoints when an individual interacts with surrounding organizations and services. The method can also systematically reflect opportunities and barriers which may influence these touchpoints during the complex process. These opportunities and barriers are then included or targeted during intervention designs based on behavior science theories.

**Fig. 1:**
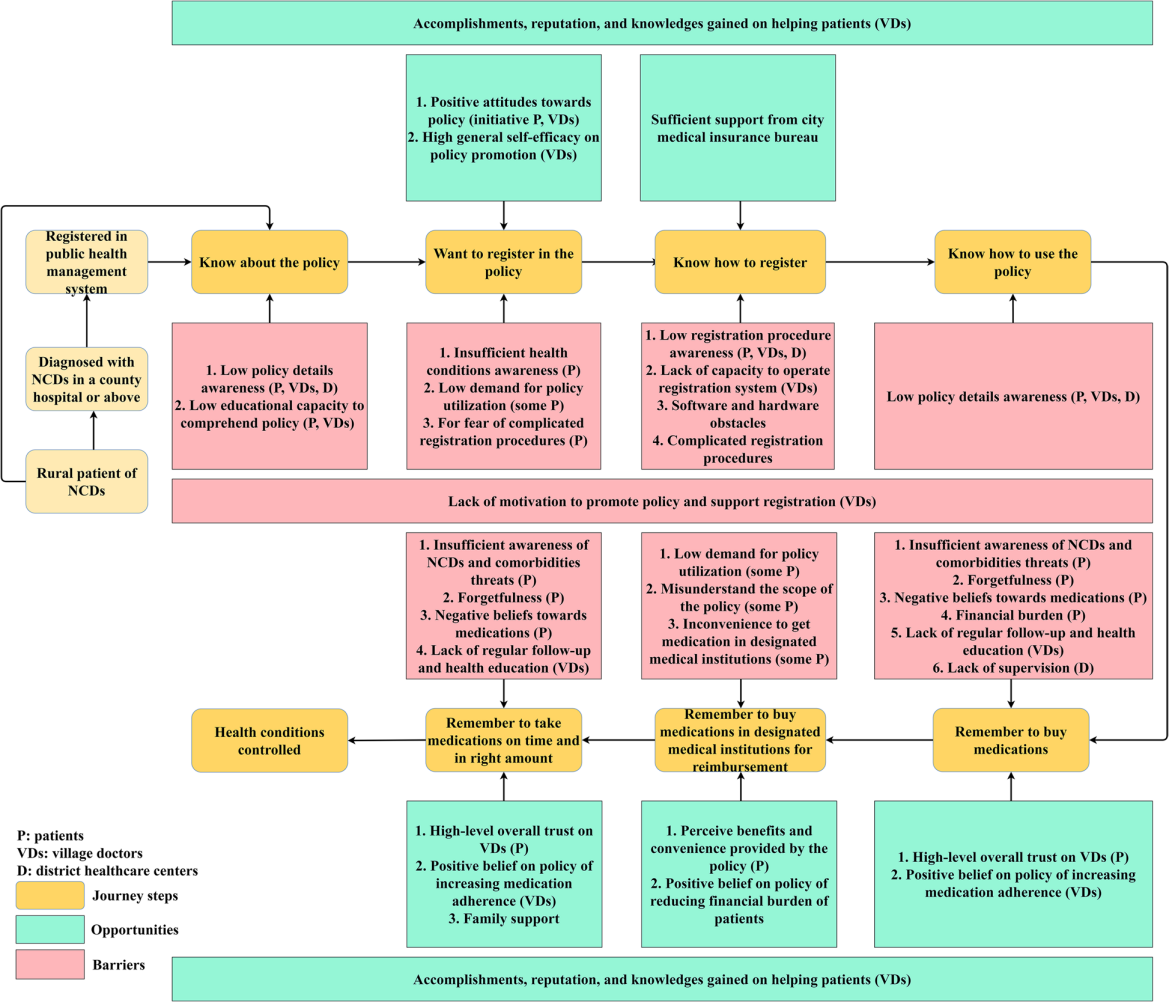
Behavior map of a rural NCDs patient’s registration in and adoption of the policy

Figure 1 shows the opportunities and barriers at each touchpoint of the patient journey in three stages—eligibility for registration, registration in village clinics or DHCs, and adoption of the policy—and at three levels: the patient level, village doctor level, and the DHC and medical insurance official level. In summary, lack of policy awareness, comprehending capabilities, positive attitudes toward diseases and medications, policy usage demand among patients, and motivation to promote the policy among village doctors are the barriers for touchpoints of the patient journey. Positive attitudes toward policy and sufficient outside support are opportunities for touchpoints of the patient journey.

#### Preliminary intervention design

The trial intervention (described in section “Intervention and control” below, Table 2, Table 3) was designed on the basis of opportunities and barriers identified in contextual research, targeted by behavior science theories [26, 31, 32, 42–46], and analyzed by the educational and ecological diagnosis of the Predisposing, Reinforcing, and Enabling Constructs in Educational/environmental Diagnosis and Evaluation and Policy, Regulatory, and Organizational Constructs in Educational and Environmental Development (PRECEDE-PROCEED) model [47–49].

**Table 2:** Barriers, opportunities, and interventions of a one-time target behavior: register in the policy system

Stakeholders	Constructs	Barriers	Opportunities	Interventions
**Patients**	**Predisposing factors**	1. Poor policy awareness 2. Insufficient health condition awareness, lack of knowledge on NCDs self-management 3. Low demand for policy utilization among some patients: (1) Acceptable outpatient medication costs (2) Used to purchase medications at pharmacies (3) Used to purchase medications by other family members	1. Initiatively consultancy with VDs on policy usage among some well-informed patients 2. Perception of policy benefits among some patients: (1) Reduce financial burden of self-medication (2) Enhance medication adherence	1. Carefully designed picture-rich posters to promote policy 2. Supplement promotion through broadcast, we-chat groups, and one-on-one promotion 3. One-on-one registration encouragement based on theories of loss aversion and encouragement 4. Encourage patients to inform policy to other patients in the village
	**Reinforcing factors**	N/A	Peer influence from registered patients	
	**Enabling factors**	Lack of mobilization from service providers	N/A	
**VDs**	**Predisposing factors**	1. Poor awareness on policy details and registration procedures 2. Low educational capacity to comprehend the policy 3. Unwilling to add additional workload on policy promotion 4. Lack of supervisions by DHCs 5. Lack of motivation	1. Possess preliminary understandings of the policy 2. Possess a positive belief on policy of helping and reducing financial burden of patients 3. High general self-efficacy on policy promotion	1. Systematic training on village doctors combined with carefully drafted training manuals and minute cards for reminder 2. Financial incentives based on performance 3. Timely report the registration progress in the communication channel 4. Clear up obstacles in the registration process by streamlining the registration procedures in DHCs and capacity building in village clinics
	**Reinforcing factors**	Lack of communication channels between VDs of different villages	1. Peer influence from initiative VDs 2. Gain positive reputation among patients 3. Gain knowledges on NCDs management	
	**Enabling factors**	1. Difficulties in social mobilization for patients, lack of policy promotion materials and language skills 2. Many elderly VDs are not capable of operating the registration system 3. Outdated hardware and software	City medical insurance bureau support hardware and software issues in primary healthcare institutions	
**DHCs**	**Predisposing factors**	1. Poor awareness on policy details 2. Lack of incentives and performance evaluation on VDs for policy promotion	1. Possess an intention to assist government on policy promotion and implementation 2. Possess an intention to reduce financial burden for patients	1. Structured communication channel between VDs and DHCs 2. Supervision and performance evaluations on VDs 3. Streamlining registration procedures through a multi-departmental coordination
	**Reinforcing factors**	Lack of communication channels with VDs	1. Better provide essential public health services for NCDs patients 2. Increase service volume	
	**Enabling factors**	1. Medical insurance staff will be overextended if large-scale registration takes place, due to high number of patients and complicated registration procedures 2. Lack of special funds for policy promotion	1. City health insurance bureau support policy promotion and implementation 2. Complicated registration process can be streamlined through coordination	

Abbreviations: *NCDs*, non-communicable diseases; *VDs*, village doctors; *N/A*, not applicable; *DHCs*, district healthcare centers

**Table 3:** Barriers, opportunities, and interventions of a recurring target behavior: adopt policy to reimburse medical expenses

Stakeholders	Constructs	Barriers	Opportunities	Interventions
**Patients**	**Predisposing factors**	1. Low demand for policy utilization among some patients: (1) Acceptable outpatient medication costs (2) Used to purchase medications at pharmacies (3) Used to purchase medications by other family members 2. Misunderstand the scope of the policy to only applying to village clinics, and chose to purchase at pharmacies due to more types of medications available 3. Lack of knowledges and belief to intake medications on time and in right amount: (1) Forgetfulness (2) Insufficient awareness of NCDs and comorbidities threats	1. High-level overall trust on VDs 2. Perceive benefits and convenience provided by policy	1. Carefully informing details of the policy to patients by VDs 2. Regular follow-up on health condition monitor, health education, and continuous reminders by VDs 3. Using calendar fliers and involving family reminders for self-management
	**Reinforcing factors**	Lack of regular reminders on adopting the policy	1. Family support 2. Peer behavior demonstration from other patients	
	**Enabling factors**	May not be convenient to get medicines at designated medical institutions for some villages	N/A	
**VDs**	**Predisposing factors**	1. Unwilling to add additional workload on large-scale patients management and follow-up 2. Medication compliance of patients is not regularly included in VD’s performance index	Possess a positive belief on policy of reducing financial burden and increasing medication adherence of patients	1. Regular follow-up on health condition monitor, health education, and continuous reminders by VDs, integrated to the regular quarterly follow-up under the essential public health services program 2. Financial incentives based on performance 3. Supervision and performance evaluation on VDs by DHCs
	**Reinforcing factors**	Lack of supervisions from DHCs	1. Gain positive reputation among patients 2. Gain knowledges on NCDs management	
	**Enabling factors**	Lack of communications and tools to remind patients adopting policy due to heavy workload	Regular quarterly follow-up visits for NCDs patients have been well established under the essential public health services program	
**DHCs**	**Reinforcing factors**	Lack of communication channels with VDs	1. Better provide essential public health services for NCDs patients 2. Increase service volume	Structured communication channel between VDs and DHCs

*Abbreviations*: *NCDs* non-communicable diseases, *VDs* village doctors, *N/A* not applicable, *DHCs* district healthcare centers

#### Pilot study

A pilot study was conducted to refine and finalize the design (described in section “Intervention and control” below). The pilot was conducted in a randomly selected village that was not included in the main trial. A training session was provided at the start of the study on policy details and registration procedures in addition to financial incentives measures and benefits for village doctors to participate in the policy promotion (Additional file 5). Four village doctors working at the village clinic attended the training session. They then promoted the policy and assisted in registration for patients based on the intervention protocol. By the end of the study, telephone interviews were conducted with village doctors to learn details of the intervention implementation and their feedback on the intervention. During the intervention period of 1 week, the registration number of NCDs patients increased from 17 to 148. Village doctors also provided positive feedback on the training manual and policy promotion posters. Insights gained from the pilot study, such as overwhelming details of the intervention and the complicated registration procedures in DHCs, were used to modify and finalize the intervention.

### Trial overview

**Fig. 2:**
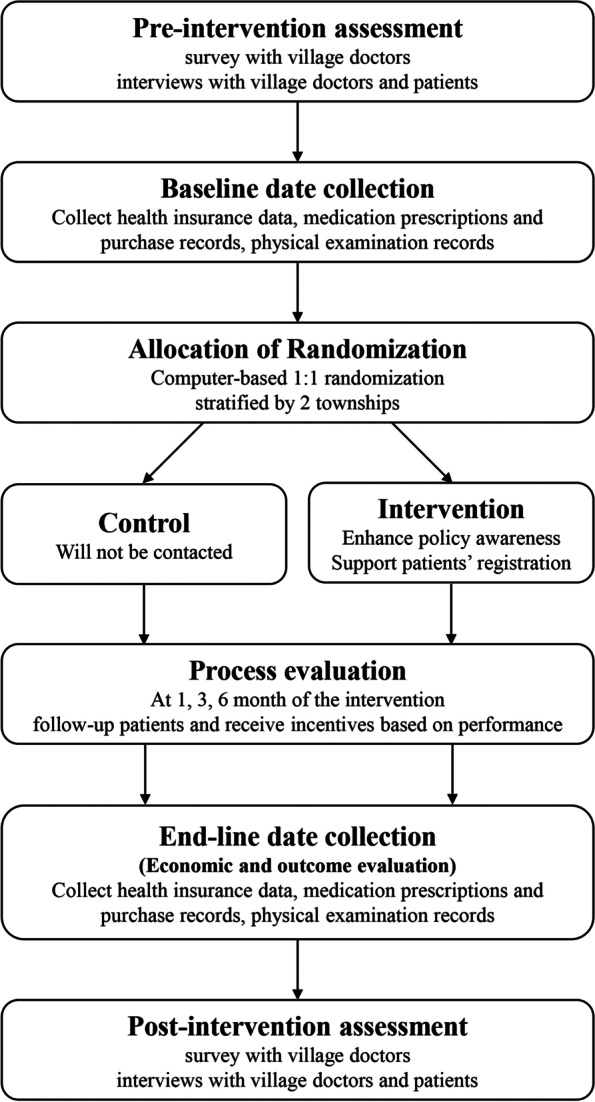
PAPMed trial flow chart

The intervention design was based on the results of the contextual research described in the previous section. PAPMed trial is an open-label cluster randomized controlled superiority trial with two parallel arms. The unit of the trial is a rural village served by a rural village clinic, which is an operational and administrative primary health clinic unit for providing NEPHS in China. All village clinics included in the trial are public-funding and non-profit institutions. Village doctors in the intervention village clinics will be main implementors of the intervention. They will promote policy, assist with registration, follow-up patients, and receive financial incentives based on their performance. Village doctors in the control group will not be contacted. The trial duration is 6 months. Figure 2 shows the trial flow chart.

### Randomization

Thirty study villages in two townships will be included in the trial. Study villages will be randomly assigned to either the intervention or control arm with an allocation ratio of 1:1. Randomization will be stratified by township to minimize township confounding factors such as differences in DHCs healthcare services. Randomization is prepared a priori by a researcher at the coordinating center, independent of trial implementation. Within each township (strata), a random number sequence based on a random number seed was generated for each village in R (R Foundation for Statistical Computing, Vienna, Austria). The numbers were then sorted in increasing order, and the first half and second half villages were labeled “A” and “B,” respectively. Finally, a virtual coin flip was performed to assign one of “A” and “B” to the intervention and control arm. Allocation will be implemented by the research team, with DHCs in both townships enrolling intervention implementors based on the allocation results. The study will be conducted as an open-label trial as it is not possible to blind the intervention implementors and rural patients. Statisticians undertaking primary analyses will be blinded to allocation.

### Study population

#### Selection of intervention implementors

Village doctors at village clinics, as well as medical insurance staffs at DHCs, are implementors of the intervention. Village doctors who participated in the trial should meet all of the following inclusion criteria: (i) employed by the local government and registered in the village doctor management system annually; (ii) participate in NCDs management services; (iii) able to communicate with patients in Mandarin or local language. Village doctors meeting any of the following criteria are excluded from the trial: (i) worked in village clinics for less than 6 months at the start of the trial; (ii) aged 75 years old or above.

For each village, the village doctor responsible for NCDs management will be selected as the coordinator and organize the implementation of the intervention with all village doctors serving in the village clinic. Medical insurance staff at DHCs will be responsible for registering patients with approved applications into the registration system. All implementors will be informed and provided written consents before participation in the study and can withdraw from the study at any time.

#### Eligibility criteria and target populations

Hypertension or diabetes patients with RBHIS meeting any one of the following criteria are eligible for registration: (i) diagnosed with hypertension or diabetes by doctors at DHCs or above; (ii) regular usage of anti-hypertensive or anti-glycemic medications, with at least 3 times of hypertension or diabetes diagnoses and medication prescriptions in medical records; (iii) hypertension or diabetes patients recorded in CDMS and follow-up by village doctors more than once [20]. Hypertension or diabetes patients with urban employee health insurance or without RBHIS are not eligible for registration.

While all rural NCDs patients meeting the eligibility criteria are potential populations of the intervention, rural NCDs patients who are recorded in the hospital information system (about 90 to 95% of all rural NCDs patients) are selected as the main targets. The small proportion of patients not in the system will be included in the study but will not be actively recruited by village doctors due to feasibility concerns.

### Intervention and control

The intervention is a behavior science-based intervention program enabling village doctors to promote policy, assist registration, and enhance medication adherence, for patients. It is designed based on the principle of being simple, feasible, and scalable, with the potential to scale up to other rural settings with similar chronic disease reimbursement schemes nationwide. A summary of the intervention with targeted opportunities and barriers is shown in Table 2 and Table 3. In brief, identified opportunities and barriers were organized for changing a one-time target behavior of registering in the policy system, and a recurring target behavior of adopting policy to reimburse medical expenses, regarding predisposing, reinforcing, and enabling constructs in the PRECEDE-PROCEED model [47], at the stakeholder levels of NCDs patients, village doctors, and DHCs. Specific interventions were designed to target each of these opportunities and barriers at different levels, and systematically integrated as an intervention program in this trial.

#### Systematic training of village doctors

The research team provided systematic training for village doctors with training manuals drafted based on governmental documents and consultations with medical insurance officials, making adjustments to fit with the knowledge level and learning capabilities of village doctors (Additional file 5). Training will include project background, details of the policy and the registration procedures, measures of the performance-based financial incentives, benefits for village doctors to participate, and details of the intervention protocol.

#### Policy promotion and registration encouragement

(i) Policy promotion: Carefully designed picture-rich posters with examples of medical costs saved per patient and contact information of responsible village doctors will be hung out in villages. Locations will be selected by village doctors and officials in village committees based on the locations’ accessibility to villagers and ability to protect the posters. These include locations such as village clinics, village committees, village activity rooms, squares, and bus stations. Village doctors are encouraged to supplement the promotion through village broadcast, We-chat groups of NCDs patients, and one-on-one promotion to target populations. Village doctors will also encourage NCDs patients to inform the policy to other patients in the village to increase policy awareness and registration based on theory of peer effects [43].

(ii) Registration encouragement: Village doctors are required to encourage main target populations to register for the policy one-on-one based on standardized structure scripts, designed by the research team based on the theories of peer effects [43], reference point [44], and loss aversion [44].

#### Clearing up obstacles in the registration process

Multi-departmental coordination consisting of medical insurance officials in the city medical insurance bureau and DHCs, and representatives of village doctors in the intervention villages is led by the research team on streamlining the registration procedures prior to the intervention. Rights and responsibilities of collecting registration materials from patients, approving registration applications, and officially registering patients in the system are clarified. Experts on the medical insurance system one-on-one trained village doctors on system operation and solving software and hardware obstacles in village clinics.

#### Inform details of the policy to patients

Village doctors are required to inform policy details to patients after registration, including the reimbursement rate and applied designated medical institutions, and to encourage using the policy. Village doctors are also required to promote medication adherence by health education on NCDs comorbidities and regular intake of medications. Patients are encouraged to use the calendar in the flyers to keep records on medication intake and to ask family members to remind them. All communications will be based on standardized structure scripts, such as “XX, a chronic disease patient in the village like you, also participated in the project,” “The annual expense reimbursement is equivalent to you selling 300 kilograms of rice,” designed by the research team based on the theories of loss aversion [44] and encouragement [45].

#### Follow-up

Village doctors are required to follow-up on all registered patients in the first, third, and sixth months after baseline and are encouraged to integrate the follow-up into regular quarterly follow-up visits under NEPHS. Village doctors will monitor blood pressure and blood glucose, provide feedback, and repeat health education for patients. Village doctors will also encourage patients to adopt the policy and intake medications on time and in the right amount. All communications will be based on standardized structure scripts, designed by the research team based on the theory of positive feedback between motivation and behavior in the self-fulfilling prophecy [46].

#### Performance feedback and incentives

Village doctors’ performances will be evaluated based on timely real-world data of policy registration and adoption, provided by DHCs. The evaluation will take place after each time’s follow-up: village doctors will receive an appropriate amount of payment based on number of patients registered in the policy after the first month follow-up and will receive the same amount of payment based on number of patients purchasing medications with reimbursement provided by the policy during the follow-up period after the third and sixth month follow-up. Design of the performance-based financial incentives to change behavior is based on nudge theory [26, 31, 32].

#### Intervention implementation

The trial management committee will be responsible for trial implementation with the coordination of local governmental officials, and officials at DHC.

- Before intervention initiation, the research team within the trial management committee will clear up obstacles in the registration process through the multi-departmental coordination aforementioned;
- The research team will educate trial implementors on details of the informed consent, and written informed consent forms (Additional file 3) will be collected from all trial implementors who voluntarily agree to participate;
- The research team will inform village doctors of intervention protocol details through systematic training at baseline. Village doctors will then promote policy, encourage registration, and inform policy details to patients based on intervention protocol;
- Within 2 weeks after baseline, the research team will one-on-one conduct surveillance of each intervention village for 2–3 rounds. The purpose is to understand the intervention implementation conditions and clear up potential obstacles;
- During the first month and 3-month follow-up, the research team will evaluate policy registration conditions and communicate with the village doctors to remind policy promotion and informing policy details to patients;
- Throughout the trial implementation, the research team will establish an online instant communication We-chat group with trial implementors and provide feedback on policy registration and adoption conditions periodically, as well as provide performance-based incentive payment for trial implementors.

#### Control

Since the trial is designed as a superiority trial to test the effectiveness of the intervention program compared to usual practices, villages in the control arm will serve as a natural control and continue their usual practice without introduction of any intervention activities. To avoid contamination of the intervention activities, village doctors in the control villages will not be contacted and will not have access to any training sessions and materials correlated to the intervention. The streamlined registration process in DHCs will only be applicable to patients from the intervention villages. Although the likelihood is small, village doctors and patients in the intervention arm will also be asked not to discuss intervention-related contents with their counterparts in the control arm. We did not anticipate and define relevant concomitant interventions that are permitted or prohibited during the trial.

### Outcome measurements

The primary outcome of the trial is the policy registration rate of NCDs patients recorded in CDMS. Information of NCDs patients registered in the policy will be collected and matched with CDMS at baseline, 3-month, and 6-month follow-up. Policy registration rate will be calculated as the proportion of NCDs patients registered in the policy in CDMS. The effectiveness of the intervention program will be evaluated by the policy registration rate between the intervention and the control arm. Secondary outcomes include the following:

- Total number of policy registrations including those that are not recorded in the hospital information system, collected at baseline and 6-month follow-up;
- Total hypertension and diabetes medication costs during duration of follow-up, collected at 6-month follow-up;
- Frequency of village doctor visits during duration of follow-up, collected at 6-month follow-up;
- Blood pressure level, including systolic blood pressure and diastolic blood pressure, measured with blood pressure monitor, if available from electronic health records or government-funded annual physical examination records;
- Blood glucose level, measured by blood tests, if available.

Data collection will rely on existing real-world administrative data (mainly insurance claims data) to reduce cost and increase study feasibility.

**Fig. 3:**
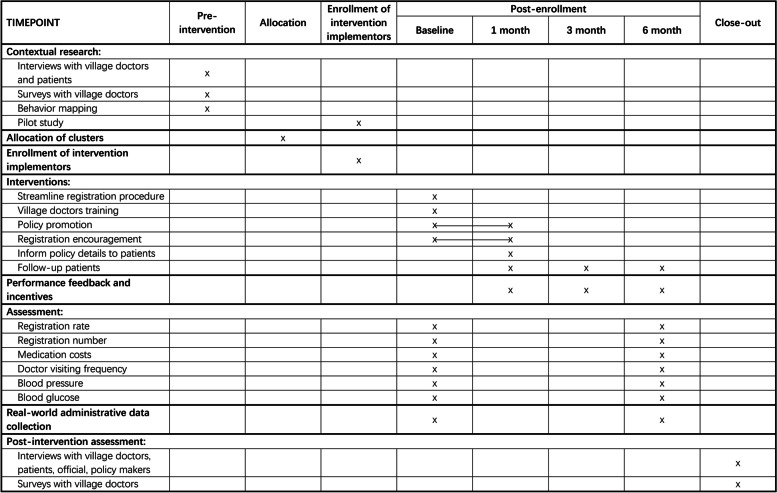
PAPMed trial Schedule of Procedures

After the end of the follow-up period, we will also collect qualitative data through in-depth individual interviews of about six village doctors, ten patients, and several government officials and policymakers. Such data will provide insight on intervention fidelity, differential effects from different intervention components, and other feedback. We will conduct post-trial assessments relying on data collection from real-world administrative data periodically, to evaluate the longer-term effects of the intervention program. We will attempt dissemination of effectiveness of the intervention program through various channels, including policy forums or other forms with local healthcare officials. The trial schedule of procedures from beginning to end is shown in Fig. 3 below.

### Analysis plan

The principle of intention-to-treat will be adopted in our analysis, where we will analyze clusters and patients as they have been randomized, regardless of the intervention they have received [50]. Individual patient data will be grouped by cluster and condensed to cluster level data. Summary analyses will be performed on a cluster level. Categorical variables will be reported as counts and proportions. Continuous variables will be reported as mean and standard deviation if symmetric or median with 25th and 75th percentile if skewed. Variables between the intervention and the control group will be compared, with *t*-test performed on continuous data and *χ*^2^ test performed on categorical data, and *P* value less than 0.05 is considered as statistically significant.

Effectiveness of the intervention on primary and secondary outcomes will be estimated with marginal modeling using generalized estimating equations (GEEs) to account for stratification and clustering [51, 52]. Robust variance estimation will be used to provide valid standard errors and to account for potential model misspecification [53, 54]. The modified Poisson approach with a log link will be used to obtain risk ratios for estimates of intervention effect with binary outcomes [53]. Main analyses will include adjustments for stratification and cluster effect. Baseline covariates will be cross-tabulated by randomization arm to check for substantial imbalance by chance. Additional analysis will be performed that will additionally adjust for identified imbalance baseline covariates. Potential effect measure modifiers impacting the effectiveness of the intervention program will be evaluated by stratified analysis. Imputation for missing data is not planned in main analyses since there is no reason to assume data is not missing at random. However, if we identify baseline covariates that are predictive of missingness, we will adjust for these covariates in additional analysis or implement multilevel multiple imputations to test for sensitivity of missing data on the results. For each outcome, we plan to report the estimate of intervention effect and its 95% confidence interval (CI), as well as estimate of intra-cluster correlation coefficients (ICCs) and its 95% CI.

### Statistical power

At the time of the contextual research and pilot study, the policy registration rate in villages of both the intervention and control arms was less than 10%. With an estimated sample of 5250 patients drawn from 15 villages in each arm and an estimated ICC of 0.04, the study is designed to provide more than 90% power (with a two-sided alpha of 0.05) to detect a difference in policy registration rate of at least 8.5% between intervention and control clusters [55, 56].

### Oversight and monitoring

The coordinating center is Duke Kunshan University and is responsible for organization of steering committee meetings. The trial steering committee consists of principal investigators in Duke Kunshan University, Nantong University, and Duke University, and is responsible for agreement on final protocol, reviewing progress of the trial, and agreement of changes to the protocol if necessary. The trial steering committee will audit the overall quality of the trial by monthly interviewing officials at DHCs and trial implementors, to confirm compliance with the requirements of the protocol. The trial management committee and data management committee consist of Duke Kunshan University and Nantong University and are responsible for study planning, coordination with governmental officials, officials at DHCs, and village doctors at village clinics, randomization, training, evaluation of trial implementors’ performance, and data management.

Any modifications to the protocol which may impact the trial conduct will require a formal amendment to the protocol, agreement by the trial steering committee meeting, and approval by the Institutional Review Board of Duke Kunshan University, prior to implementation. Trial implementors will be formally notified about such modifications by the trial management committee under coordination with governmental officials and officials at DHCs. Any modifications to the protocol impacting rights related to trial implementors will require supplementary signed informed consent under surveillance of the trial steering committee and the Institutional Review Board of Duke Kunshan University.

### Data management and confidentiality

The data management committee will oversee the data-sharing process. All identifiable information (such as informed consent forms and insurance claims data of patients) will be electronically stored on a password-protected hard drive under the control of the coordinating center with access only to principal investigator and trial manager at the coordinating center. Hard copies will be kept in locked file cabinets in the coordinating center with access only available to the trial manager. Participants’ information will not be released outside of the study without the written permission of the participant. All principal investigators in the trial steering committee will be given access to de-identified cleaned condensed cluster-level data sets.

## Discussion

This research is a novel study that promotes the utilization of outpatient reimbursement policies for chronic diseases through behavioral intervention. The PAPMed trial is innovative as it sorts out the barriers and facilitators in the policy implementation in rural areas, builds a simplified registration process at township and village levels, improves the social mobilization capacity of primary healthcare workers in the rural area, and designs the strategies to effectively promote the use of this policy by village doctors and rural patients. The findings of the study will provide translational evidence for other resource-constrained settings in developing strategies for the utilization of outpatient medicine reimbursement policies for chronic diseases.

In the contextual research phase, the barriers and opportunities in policy implementation among multi-stakeholders were identified through literature reviews and surveys in study sites and organized through behavior mapping. The predisposing factors, enabling factors, and reinforcing factors of multiple stakeholders are identified by the educational and ecological diagnosis of PRECEDE-PROCEED model. Several key insights have been found. For the patients, there is no established channel for patients to become aware of the reimbursement program. For the village doctors, the lack of motivation and the complexity of the operational process inhibits effective implementation and adoption of the reimbursement program.

The principles of intervention design are simple, feasible, and scalable. The design of the PAPMed intervention was informed by behavioral theories and field-based contextual research to support village doctors. Firstly, the registration procedure will be simplified (a clear visualization flow chart of the steps of record and a streamlined recording procedure between THCs and village doctors through multi-departmental coordination will be established). Secondly, competence training for village doctors is designed to help them carry out their daily work in the local primary healthcare system. Thirdly, although the results of existing studies on performance payment for primary healthcare doctors to provide healthcare services are not consistent [57–59], a performance-based payment and a series of nudge strategies will be offered to village doctors in this project. The performance-based payment program will be based on the number of patients recorded and the number of patients purchasing medications at designated medical institutions for reimbursement in the program. Under the premise of certain benefits, doctors will work hard to increase the registration and utilization rate of patients.

We adopted several behavioral theories to address barriers identified on the patient side. Nudge theories and strategies have been used in health behavior intervention for patients with chronic diseases [60]. In this study, nudging elements such as reminder, framing, social modeling, and social norm were applied to the design of intervention strategies at the patient level, which mainly includes carefully designed picture-rich posters and calendar fliers to promote policy and regular follow-ups to supervise use of the policy and medication adherence by village doctors. The focus is to help patients perceive losses from not using the policy and social norms, in addition to peer influence through the comparison of medication compliance with other patients.

We acknowledge several limitations in our study. First, due to limited funding, the study is planned to be carried out only in a single county, affecting the generalizability of the study. However, the intervention and data collection are designed to be simple, feasible, and scalable and is expected to have the capability to adapt to similar settings. Second, due to lacking the records of NCDs patients who are not recorded in the hospital information system, we did not target this group of patients. But these patients constitute a small proportion (approximately 5 to 10%) and are mostly immigrant residents to other regions of the country, thus do not have access to local policies inherently. Third, the intervention period will last for only 6 months which may hinder observation of full policy effects.

Since 2019–2020, the national and local medical insurance bureaus have issued new chronic disease outpatient reimbursement policies [17–19]. At present, there are no studies and reports on the implementation of this policy in various regions. We identified the barriers and facilitators of stakeholders’ behavior in the policy implementation. The registration process will be streamlined at the system level to address these barriers. Intervention strategies designed based on behavioral change theories such as nudging, performance-based incentives, and mobilization toolkits will be provided to village doctors. Additionally, necessary medication adherence reminders will be provided for patients. The findings of this trial will not only provide evidence on the feasibility and effectiveness of the intervention but may also inform policy implementation for other low-income regions where outpatient medical costs remain a financial burden for patients.

### Trial status

Protocol version 2021-01-29. Recruitment start date: 2021-01-30. Estimated completion date: 2021-09-30.

## Supplementary Information

**Additional file 1.**  Essentials of Nantong City’s Policy on the Reimbursement of Chronic Disease Medicines. Translations of essentials of Nantong City local governmental policy, “Issuance of the Implementation Rules for the Management of Outpatient Medication for Hypertension and Diabetes in Nantong Resident Medical Insurance”.

**Additional file 2.**  PAPMed trial registration data. Detailed data on PAPMed trial registration in clinicaltrials.gov and Chinese Clinical Trial Registry.

**Additional file 3.**  Written consent form. Written consent form for participated village doctors.

**Additional file 4.**  SPIRIT checklist.

**Additional file 5.**  Essentials of Training Manuals for Village Doctors in Intervention Villages. Translations of training manuals essentials for systematic training on village doctors in intervention villages.

## Data Availability

Not applicable

## Abbreviations

PAPMed: Promoting the adoption of local governmental policy on the reimbursement of chronic disease medicines
NCDs: Non-communicable diseases
RBHIS: Residents-based Basic Health Insurance Schemes
RMB: Ren Min Bi
CDMS: Chronic disease management system
THCs: Township health centers
CHWs: Community health workers
NEPHS: National Essential Public Health Services
SPIRIT: Standard Protocol Items: Recommendations for Interventional Trials
USD: United States Dollar
DHCs: District health centers
PRECEDE-PROCEED: Predisposing, Reinforcing, and Enabling Constructs in Educational/environmental Diagnosis and Evaluation and Policy, Regulatory, and Organizational Constructs in Educational and Environmental Development
GEEs: Generalized estimating equations
CI: Confidence interval
ICCs: Intra-cluster correlation coefficients
