# Supplementary material for: Promoting the adoption of local governmental policy on the reimbursement of chronic disease medicines (PAPMed): study protocol of a field-based cluster randomized trial in rural Nantong, China
Source: Trials. 2022 Sep 15;23:785. doi: 10.1186/s13063-022-06710-1 (PMC9479297; doi:10.1186/s13063-022-06710-1)
Supplement: Supplementary file 1 — Additional file 1. Essentials of Nantong City’s Policy on the Reimbursement of Chronic Disease Medicines. Translations of essentials of Nantong City local governmental policy, “Issuance of the Implementation Rules for the Management of Outpatient Medication for Hypertension and Diabetes in Nantong Resident Medical Insurance”. [file 13063_2022_6710_MOESM1_ESM.docx]

**Additional File 1. Essentials of Nantong City’s Policy on the Reimbursement of Chronic Disease Medicines**

**Nantong Healthcare Security Administration's and Nantong Health Commission's notice on "Issuance of the Implementation Rules for the Management of Outpatient Medication for Hypertension and Diabetes in Nantong Resident Medical Insurance" ***

1. Eligibility criterion for hypertensive and diabetic patients
   1. Eligibility criterion for hypertensive patients: meeting any one of the following criteria are eligible for registration
      1. Systolic blood pressure ≥ 140 mmHg and/or diastolic blood pressure ≥ 90 mmHg, measured at rest for three times in different days, and need medication treatments;
      2. Regular usage of anti-hypertensive medications, with at least 3 times of hypertension diagnoses and medication prescriptions in medical records.
   2. Eligibility criterion for diabetic patients: meeting any one of the following criteria are eligible for registration
      1. Diabetes symptoms (typical symptoms include thirst, excessive drinking, polyuria, and weight loss for unknown reasons) and at least one of the following:
         - Plasma glucose ≥ 11.1 mmol/L (200 mg/dl) at any timepoint;
         - Plasma glucose ≥ 7.0 mmol/L (126 mg/dl) when fasting (fasting time > 8 hours);
         - Plasma glucose ≥ 11.1 mmol/L (200 mg/dl) 2 hours after 75g glucose load;
      2. At least two of 1), 2), and 3) without diabetes symptoms;
      3. Regular usage of anti-glycemic medications, with at least 3 times of diabetes diagnoses and medication prescriptions in medical records.
2. Medical institutions for confirming hypertension and diabetes diagnosis

- City-wide designated hospitals with the qualification of diagnosis and treatment of hypertension and diabetes at the second level and above.

1. Medical institutions for hypertension and diabetes medication services

- Citywide designated medical institutions with medical service scope for outpatient chronic diseases that meet the regulations of credit rating management of medical insurance integrity services.

1. Ways of registration to the policy for hypertensive and diabetic patients
   1. Previous diagnosed hypertensive and diabetic patients: Based on hospital information system, for patients with clearly diagnosed hypertensive and diabetic, after more than one face-to-face confirmation, continuously taking medications for treatment, and participating in health insurance, they can register in the policy via personal application and get approval for registration in designated township health center (village health room) or community health service center (station).
   2. Newly diagnosed hypertensive and diabetic patients: Patients who are newly diagnosed with hypertension or (and) diabetes are eligible for medication reimbursement after they have been diagnosed, apply and ger approval for registration at designated hospitals.
2. Hypertension and diabetes outpatient medication reimbursement coverage

- From the date of registration, hypertensive and diabetic patients are eligible for reimbursement of up to 1600 Ren Min Bi per person per year for patients with hypertension or diabetes disease and 2000 Ren Min Bi per person per year for patients with both diseases, for outpatient drug expenses incurred in the designated medical institutions in accordance with the provisions of medical insurance. The medication fee will be paid by the social medical fund at a rate of 50%.

Reference:

* Nantong Healthcare Security Administration. Nantong Healthcare Security Administration's and Nantong Health Commission's notice on "Issuance of the Implementation Rules for the Management of Outpatient Medication for Hypertension and Diabetes in Nantong Resident Medical Insurance". 2019.
