# Supplementary material for: Promoting the adoption of local governmental policy on the reimbursement of chronic disease medicines (PAPMed): study protocol of a field-based cluster randomized trial in rural Nantong, China
Source: Trials. 2022 Sep 15;23:785. doi: 10.1186/s13063-022-06710-1 (PMC9479297; doi:10.1186/s13063-022-06710-1)
Supplement: Supplementary file 2 — Additional file 2. PAPMed trial registration data. Detailed data on PAPMed trial registration in clinicaltrials.gov and Chinese Clinical Trial Registry. [file 13063_2022_6710_MOESM2_ESM.docx]

**Additional File 2. PAPMed trial registration data**

| Data category | Information |
| --- | --- |
| Registry and trial identifying number | ClinicalTrials.gov (NCT04731194)  Chinese Clinical Trial Registry (ChiCTR2100042152) |
| Date of registration in registry | ClinicalTrials.gov: January 29, 2021  Chinese Clinical Trial Registry: January 14, 2021 |
| Sponsor | Duke Kunshan University |
| Contact for public queries | Lijing Yan, PhD, MPH,  lijing.yan@duke.edu  Yuexia Gao PhD 386912453@qq.com |
| Contact for scientific queries | Lijing Yan, PhD, MPH,  lijing.yan@duke.edu  Yuexia Gao PhD 386912453@qq.com |
| Public title | Promoting Medication Reimbursement Policy (PAPMed) |
| Scientific title | Promoting the Adoption of Local Government Policy on the Reimbursement of Chronic Disease Medicines (PAPMed): a Field-based Cluster-Randomized Controlled Trial in Rural Nantong, China |
| Countries of recruitment | China |
| Health condition(s) or problem(s) studies | local government policy on the reimbursement of chronic disease medicines |
| Intervention(s) | Behavioral: Performance-based financial incentive program  1. Enhance policy awareness through verbal communication with patients, distributing picture-rich fliers to patients, and putting up large posters in public places of the villages.  2. Support patients' registration in the reimbursement system  3. Follow-up patients after the first, third, and sixth months after the start of the study, measuring blood pressure and blood glucose level. Encourage patients to purchase medications from public institutions and to take their medications on time.  4. Receive financial incentives based on performance in the amount of 3 RMB per patient enrolled (first month) and 5 RMB per patient (at third and sixth month) buying medications with reimbursement from the policy. |
| Key inclusion and exclusion criteria | Inclusion criteria:  To be eligible to register in the medication reimbursement policy, patients need to be:  1. Living in the service areas of the village clinics  2. Officially diagnosed with hypertension and/or diabetes in a township level hospital or above  3. Registered as a hypertensive and/or diabetic patient in the public health service system |
|  | Exclusion criteria:  Not part of the New Cooperative Medical Scheme (NCMS) for rural residents |
| Study type | Parrel assignment, open label, cluster randomized controlled trial |
| Target sample size | 5000 |
| Primary outcome(s) | Registration number (time frame: 6 months) |
| Key secondary outcomes | 1. Registration number (time frame: 6 months)  2. Medical costs saved (time frame: 6 months)  3. Doctor visiting frequency (time frame: 6 months)  4. Medication compliance rate (time frame: 6 months)  5. Blood pressure (time frame: 6 months)  6. Blood glucose (time frame: 6 months)  7. Blood lipids (time frame: 6 months) |
