# Supplementary material for: Promoting the adoption of local governmental policy on the reimbursement of chronic disease medicines (PAPMed): study protocol of a field-based cluster randomized trial in rural Nantong, China
Source: Trials. 2022 Sep 15;23:785. doi: 10.1186/s13063-022-06710-1 (PMC9479297; doi:10.1186/s13063-022-06710-1)
Supplement: Supplementary file 3 — Additional file 3. Written consent form. Written consent form for participated village doctors. [file 13063_2022_6710_MOESM3_ESM.docx]

**Additional File 3. Written Consent Form**

**Consent Form**

**Note: The consent process will be conducted by collaborators whose IRB will cover their activities.**

**The translation of the Chinese version has centered on the principle of being comprehensible to the participants.**

**Study name**: **Promoting the adoption of local government policy on the reimbursement of chronic disease medicines—a field experiment in rural Nantong, China**

**Abbreviation: PAPMed**

Dear Sir or Madam:

This consent form describes the research study to help you decide if you want to participate. Please read the following information carefully and decide voluntarily whether you would like to participate in this study.

**What is the purpose of this study?**

Efforts need to be made to address the heavy economic burden of chronic diseases, particularly in the elderly in rural areas. Nantong City has recently established a reimbursement plan which covers 50% of hypertension and diabetes medication costs. However, this policy is in the newly born stage and many of the rural citizens with chronic diseases are uninformed of this reimbursement scheme thus might lower its positive effects on reducing the financial burdens of the patients. Even patients that are aware of this policy might not be able to take advantage of it due to many obstacles. We hope to promote this scheme to rural patients through informative and educational approaches to decrease medical costs and increase medicine compliance rates.

**Must I participate in this study?**

Participation in this study is entirely voluntary. You are free to withdraw at any stage of the study.

**What will happen during this study?**

Using villages as clusters, we plan to adopt a cluster randomized controlled approach and only include one village under each village clinic’s administration. After getting consent from you, you will perform the intervention lasting half a year to promote policy adoption. The detailed tasks are provided below.

**What are the risks of this study?**

There is no or minimal risk of participating in this study.

**What are the benefits of this study?**

You will gain more knowledges on hypertension and diabetes management, enabling you to better serve their patients. You may gain an additional positive reputation from helping patients to save money.

**Incentive structure**

You will be provided with incentives after the first, third, and sixth-month follow-up visits, respectively. The first incentive is based on the number of patients contracted in the first month, and you will be given five yuan per contract; the second incentive is you will be rewarded 5 yuan per patient by the number of patients who have purchased discounted medicines in the first three months; the third incentive is you will be rewarded 5 yuan per patient by the number of patients who have purchased discounted medicines during months 3-6.

**Other important information**

This study will abide by the standard of Good clinical practice of ICH and ethical principles based on the Declaration of Helsinki.

This study has been approved by the ethics committee of Duke Kunshan University and Nantong University.

**What are your rights?**

Your participation in this study is completely free and voluntary. You are free to withdraw for any reason at any stage of the study.

**What do you need to do to participate in this study?**

If you are allocated to the intervention group, you will have the following responsibilities:

1. At the cluster level: posting policy advertising posters in village clinics, village councils, activity rooms, and other places frequented by residents. Posters are carefully designed and repeatedly delivered at multiple points based on the principles of behavioral science and audience characteristics.

2. At the individual level:

(a) One-to-one awareness-raising on the benefits of the policy and distribution of leaflets on the policy.

(b) Encourages and assists patients to register in the drug reimbursement policy.

(c) Follow up patients after the first month, third month and sixth month after the start of the study, measuring blood pressure and blood glucose level. Encourage patients to purchase medications from public institutions and to take their medications on time. The first month can be a telephone follow-up, and the 3rd and 6th-month follow-ups are recommended to be combined with quarterly screenings.

**How will your privacy and personal sensitive information be protected?**

This is a cross-institutional collaborative research between Nantong University (Lead investigator: Prof. Yuexia Gao), Duke Kunshan University (Lead investigator: Prof. Lijing Yan). The researchers in charge of this study will record some of your personal information, including your names, phone numbers, date of birth, and other necessary data. After village doctor’s recruitment, all identifiable information will be transmitted through password-protected secure channels and electronically stored on a password-protected hard drive under the control of DKU IT with access only to PI and project manager. Data will be stored in two forms: the identifiable dataset and coded dataset. The identifiable dataset will be kept in a password-protected hard drive in the DKU global health research center with the access only available to the project manager and PI. The coded (de-identified) dataset will not have any personally identifiable information and will be used for research analysis purposes. It will be stored in separate files with password protection under the DKU protected network. The identifiable dataset will not be used except in circumstances when necessary such as during quality control.

**Who will bear the cost of this study?**

All related costs and payment of this study will be shouldered by the researchers.

**Thanks for your participation and support!**

**Informed Consent Form**

I agree to participate in this study by signing this form.

I volunteer to participate in this study, not impacted by any interest commitment or other aspects like economy nor forced by the researcher.

I am aware that I can withdraw from this study at any stage.

I understand everything about the introduction and explanation of this study, and I have enough time to consider my participation.

In view of the current privacy law and sensitive information, as described in the part of subject information, I agree that researchers, after taking full accounts of my rights, can have access to my data.

I confirm that I have obtained a copy of this informed consent form.

Signature of participant_______________________ Date _________________

or

Signature of relatives of participant _______________________ Date _________________

**Investigator’s declaration**

I declare that I have provided participants with complete and detailed information on the characteristics, purpose, procedure, and duration of this trial.

I declare that I have provided the participants with their information and a copy of the informed consent form with signature and date.

Signature of investigator ________________________ Date ______________________

Name of investigator (capital letters) _______________________________________
