# Supplementary material for: Promoting the adoption of local governmental policy on the reimbursement of chronic disease medicines (PAPMed): study protocol of a field-based cluster randomized trial in rural Nantong, China
Source: Trials. 2022 Sep 15;23:785. doi: 10.1186/s13063-022-06710-1 (PMC9479297; doi:10.1186/s13063-022-06710-1)
Supplement: Supplementary file 5 — Additional file 5. Essentials of Training Manuals for Village Doctors in Intervention Villages. Translations of training manuals essentials for systematic training on village doctors in intervention villages. [file 13063_2022_6710_MOESM5_ESM.docx]

**Additional File 5. Essentials of Training Manuals for Village Doctors in Intervention Villages**

- **Policy core**
  - **Target population:**
    - Rural residents who covered by basic health insurance.
    - Diagnosed with hypertension or diabetes by doctors at DHCs or above.
    - Took hypertension or diabetes medicines and have more than three-time prescriptions with hypertension or diabetes medicines in out-patients or inpatients medical records.
    - Hypertension or diabetes patients registered in Chronic Diseases Management System.
  - **Policy treatment:** This policy covers up to 1600 RMB for patients with hypertension or diabetes and 2000 RMB for patients with both diseases annually.
  - **Service institutions:** Authorized medical institutes that provide outpatient special medical services for chronic diseases.
  - **Scope of payment:** Within the purview of medical insurance, it can be used to cover the cost of medicine, insulin, and examinations for common and chronic diseases.
- **Benefits of village doctors**
  - **Gain an additional positive reputation from helping patients to save money.**
  - **Improve village doctors’ medical knowledge and skills.**
  - **Increase income for village doctors and obtain economic incentives.**
- **Content of the work**
  - **Post policy posters** in prominent locations like village clinics and committees. Supplemented by **village broadcasting and WeChat groups** to promote the policy to residents.
  - Inform patients about the benefits of the policy and provide a policy publicity calendar.
  - **Encourage and help** patients to participate in medication reimbursement policy.
  - **Remind** patients to use the medication reimbursement policy to buy medicines from designated institutions and take medicines as prescribed by village doctors.
  - **Follow up** with the registered patients (the first, third, and sixth months of registration). Financial incentives for village doctors according to the number of registered patients and the number of patients who use the policy to buy medicine after registration.
- Follow-up suggestions
  - **Scripts for mobilization.** “You will save at least 1000 yuan per year by using this policy, which is equivalent to selling 1000 eggs.”
  - **Scripts for registration.** “Bring your health insurance card and records to DHCs and you will be registered. After that, you will be able to use the benefits of the policy. We suggest that you come to our village clinic once a month to get your medication. Please contact me if you have any questions.”
  - **Scripts for follow-up.** “You are welcome to come here to get medicine

any time after registration.” “Medicare is not reimbursed when you buy medicine at private pharmacies.” “Every time you come to get medicine, please mark a circle on your calendar to remind yourself that you have taken your medicine on time. Gradually, you will develop regular medication habits. If you are healthy, it is good for your family and our community.”

- - You can remind your patients about using this policy frequently during the routine chronic disease follow-up.
  - Show your patients enough patience, understanding and respect.
